# SLC25A19 drives colorectal cancer progression by regulating p53

**DOI:** 10.1002/cam4.70253

**Published:** 2024-09-30

**Authors:** Jinbo Jiang, Xuemei Li, Jiayong Wang, Shaofei Chen, Lingjuan Chen

**Affiliations:** ^1^ Department of General Surgery, Qilu Hospital Shandong University Jinan Shandong China; ^2^ Advanced Medical Research Institute, School of Medicine, Shandong University Jinan Shandong China; ^3^ Department of Gastrointestinal Surgery, Union Hospital, Tongji Medical College Huazhong University of Science and Technology Wuhan China; ^4^ Cancer Center, Union Hospital, Tongji Medical College Huazhong University of Science and Technology Wuhan China; ^5^ Institute of Radiation Oncology, Union Hospital, Tongji Medical College, Huazhong University of Science and Technology Wuhan China

**Keywords:** apoptosis, colorectal cancer, p53, proliferation, SLC25A19

## Abstract

**Background:**

Investigating the molecular mechanism of colorectal cancer (CRC), a common lethal malignancies worldwide, is of great clinical significance. Solute carrier family 25 member 19 (SLC25A19) is a member of the solute carrier family that contribute to cellular functions, including tumor biology. Recently, many studies have attention on uncovering the relationship of SLC25A19 with malignant cancers, but its precise involvement in the regulation of CRC has not been thoroughly understood. This study sought to uncover the role and mechanism of SLC25A19 in CRC development.

**Methods:**

The GEPIA database and immunohistochemical staining were utilized to detect the expression of SLC25A19 in CRC tissues. The functional influences of SLC25A19 on CRC cell phenotypes were evaluated through a series of assays including celigo cell count, colony formation, CCK‐8, flow cytometry, wound healing, and transwell assays following knocking down SLC25A19. Subsequently, the xenograft tumor model was constructed to evaluate the effect of SLC25A19 on tumor growth in vivo. The underlying mechanisms of SLC25A19 silencing were investigated using the human phospho‐kinase array.

**Results:**

This study demonstrated the upregulation of SLC25A19 in CRC and its significant correlation with unfavorable prognosis in CRC patients. Suppression of SLC25A19 resulted in significant inhibition of cell proliferation, colony formation, and cell migration, alongside a boost in cell apoptosis. In vivo experiments revealed that silenced SLC25A19 displayed reduced growth rates and formed smaller xenografts. Mechanistically, the p53 pathway was found to be upregulated by SLC25A19 knockdown and mediated the function of SLC25A19.

**Conclusions:**

Consequently, SLC25A19 was identified as a novel molecule with key regulatory ability in CRC development.

## 1 INTRODUCTION

Colorectal cancer (CRC) is a gastrointestinal tract malignancy that typically exhibits asymptomatic or insignificant symptoms during its early stages. It ranks as the third most prevalent cancer worldwide.^1^ In 2020, the World Health Organization (WHO) reported almost 1.93 million new cases and 916,000 deaths from CRC (https://www.who.int/news‐room/fact‐sheets/detail/cancer). Generally, surgery is considered the gold standard for treating CRC and has improved the survival of CRC patients. However, many CRC patients are extremely prone to liver metastasis, causing secondary damage to the body and increasing the difficulty of treatment.^2^, ^3^ Although chemotherapy and radiation therapy have improved clinical outcomes, chemoresistance and recurrence frequently pose a major challenge.^4^ Researching molecular mechanisms is currently one of the most exciting areas within tumor‐related research, offering new potential targets and treatment avenues for tackling tumors.^5^, ^6^, ^7^ However, due to a limited understanding of the complex molecular mechanisms involved in CRC development, targeted therapies for CRC have faced obstacles at a bottleneck stage.^8^ Therefore, elucidating the molecular mechanisms underlying CRC development and identifying biomarkers for early diagnosis and prognostic assessment are crucial for its treatment.

Solute carrier family 25 member 19 (SLC25A19) belongs to the solute carrier family, responsible for encoding the specific thiamine mitochondrial carrier, which is of critical importance to various metabolic activities within cells occurring in the cytosol, mitochondria, and peroxisomes, ultimately influencing mitochondrial functions.^9^, ^10^ Moreover, SLC25A19 likely participates in alterations of pyruvate metabolism and has been found to be specifically mutated in human metabolic syndromes.^11^ Previous studies have indicated that loss of SLC25A19 causes embryonic lethality, malformations of the central nervous system, and anemia.^12^, ^13^ Notably, there have been reports indicating the overexpression of SLC25A19 in breast cancer.^14^ Moreover, SLC25A19 was identified as a gene associated with artesunate sensitivity and resistance in clinical oncology.^15^ Thus, SLC25A19 has attracted our greatest attention as a potential therapeutic target. Nevertheless, the expression and exact functional roles of SLC25A19 in CRC are still entirely unknown and need further investigation.

The objective of this study was to explore the involvement of SLC25A19 in CRC and its mechanism in regulating of cancer progression. Initially, we focused on analyzing the expression of SLC25A19 and its correlation with clinical characteristics and prognosis in CRC patients. Additionally, we delved into the functional roles of SLC25A19 knockdown at the cellular level and in animal models. Subsequent mechanism study revealed that SLC25A19 modulated cellular processes via regulating the p53. In summary, our findings underscore the significance of SLC25A19 in CRC pathogenesis and provide insights into its potential as a therapeutic target.

## 2 MATERIALS AND METHODS

### 2.1 Bioinformatics analysis

Expression data for SLC25A19 in colorectal adenocarcinoma (COAD), including microsatellite instability‐high (MSI‐H), microsatellite instability‐low/microsatellite stable (MSI‐L/MSS) subtypes, and normal samples, were sourced from The Cancer Genome Atlas (TCGA) and the Genotype‐Tissue Expression (GTEx) project. This data was obtained in a processed form directly from the Gene Expression Profiling Interactive Analysis (GEPIA) 2 platform to ensure consistency and reliability. Strict thresholds were set at |log2 fold change (FC)|>1 and a *p* value <0.01.

### 2.2 Cell and tissue microarray

Human CRC cells (HCT 116, RKO, SW480, HT29, and DLD‐1) and human normal colonic epithelial cells (FHC) were sourced from ATCC. SW480 cells were cultured in L‐15 medium containing 10% FBS, while HCT 116, RKO, DLD‐1, and FHC cell lines were grown in RPMI 1640 with 10% FBS. HT29 cells were cultured in McCoy's 5A medium modified supplemented with 10% FBS. The cells were all maintained at 37°C in a humidified atmosphere containing 5% CO_2_. In some experiments, cells were treated with 20 μM pifithrin‐α (PFTα; S2929, Selleck, USA) or left untreated.

A tissue microarray, which harbored 98 paired CRC tissues and 103 paired para‐carcinoma tissues, was purchased from Shanghai Yibeirui Biosciences (YBR‐HCol119‐M002, Shanghai, China). Patient consent was obtained, and all human studies adhered to the principles of the Helsinki Declaration. Clinical parameters recorded included age, gender, lymph node status, lymph node invasion, tumor differentiation, stage, tumor infiltration, lymphatic metastasis, vascular invasion and metastasis. The average age of the patients, comprising 57 males and 41 females, was 61 years (range 31–91 years). Cancer stages were recorded as follows: stage 1 (*n* = 11), stage 2 (*n* = 36), stage 3 (*n* = 43), and stage 4 (*n* = 8). The association between SLC25A19 expression and various clinicopathological features was assessed for categorical variables.

### 2.3 Immunohistochemical (IHC) staining

Tissue sections embedded in paraffin were treated with xylene and dehydrated using a gradient of ethanol solutions. Subsequently, the samples were subjected to antigen retrieval in citric acid buffer, followed by blocking endogenous peroxidase activity with 5% H_2_O_2_ and nonspecific binding sites with 5% serum for 15 min. After that, the sections were cultured overnight at 4°C with the primary antibodies anti‐SLC25A19 (1:100, ab19022, Abcam) or Ki67 antibody (1:300, ab16667, Abcam), and subsequently treated with the secondary antibody goat anti‐rabbit IgG H&L (HRP) (1:400, ab97080, Abcam) for 1 h at room temperature. Following washing with 1 × PBST, the sections were darkly stained in DAB staining solution for 5 min and then counterstained with hematoxylin for 10–15 s, followed by observed and photographed through a microscope. The proportions of positive cells were calculated under five randomly selected high‐power fields per tissue section by two independent, blinded observers. IHC scoring of the specimens was classified as negative (0), positive (1–4), ++ positive (5–8), or +++ positive (9–12), based on the sum of the staining intensity and staining extent scores. Patients with CRC were categorized into two groups based on SLC25A19 expression: low expression (*n* = 52) and high expression (*n* = 46), with the median value of 4 serving as the cutoff (high expression > median, low expression ≤ median).

### 2.4 Establishing stable cell lines with SLC25A19 knockdown

Short hairpin RNA (shRNA) sequences targeting SLC25A19 (shSLC25A19‐1: 5′‐CTCGTATGAATTCTTCTGTAA‐3′; shSLC25A19‐2: 5′‐TACGGAGATACAAGGGCCTCA‐3′; shSLC25A19‐3: 5′‐TTCTCAGTGCACTTTGTATGT‐3′) and negative shRNA sequences (shCtrl: 5′‐TTCTCCGAACGTGTCACGT‐3′) were commercially synthesized by Shanghai Sangon Biotechnology Co. Ltd. In brief, shRNAs were ligated into digested BR‐V108 lentivirus vectors, annealed to form double‐stranded DNA, and inserted into Escherichia coli TOP10 competent cells for amplification. Subsequently, the modified plasmids were grown in LB liquid medium supplemented with 100 μg/mL ampicillin (Amp), purified using an Endofree Plasmid Maxi kit (Qiagen), and then co‐transfected into 293 T cells along with the viral packaging helper plasmid, followed by incubation at 37°C for 48 h. Subsequently, the supernatants were harvested and centrifuged to isolate the virus particles. Following quality assessment, these virus particles were used to transfect RKO and HCT 116 cell lines for 48 h, and their transfection efficiency was evaluated under a microscope.

### 2.5 Real‐time quantitative PCR detection system (qPCR)

Cellular RNA was extracted with TRIzol Reagent (Invitrogen) following the manufacturer's guidelines, and the concentration and purity of the RNA were assessed via spectrophotometry. Subsequently, total RNA underwent reverse transcription to synthesize cDNA using the Promega M‐MLV kit. The PCR system was prepared with SYBR Premix Ex Taq mix after cDNA purification. Real‐time PCR was conducted using a Vii7 Real‐time PCR System (ABI Company, USA) in a two‐step procedure. The cycling conditions were as follows: initial denaturation at 95°C for 5 min, followed by 40 cycles of denaturation at 95°C for 30 s, annealing at 60°C for 30 s, and extension at 72°C for 30 s. Each sample was analyzed in technical triplicates. Specificity of the amplification was confirmed through melting curve analysis post‐amplification. 2^−ΔΔCt^ was used to calculate the mRNA level of genes. The experiment employed specific forward and reverse primers, including SLC25A19 forward primer (5′–CAGATGGCAGGAATAACACC‐3′, reverse primer 5′–ATGCTGAAGCTGGAAACG‐3′) and GAPDH forward primer (5′–TGACTTCAACAGCGACACCCA‐3′, reverse primer 5′‐CACCCTGTTGCTGTAGCCAAA‐3′).

### 2.6 Western blot

Cellular protein was obtained by extracting with RIPA protein lysate, and the protein concentration was determined using the BCA Protein Assay Kit from HyClone‐Pierce. Next, 20 μg of the protein underwent SDS‐PAGE electrophoresis and was transferred to PVDF membranes. The membranes were then treated with 1 × TBST solution containing 5% skimmed milk for 2 h at room temperature, followed by a 2‐h incubation with the primary antibody. After wishing, the secondary antibody was subsequently added and incubated for an additional hour at room temperature. Finally, the protein bands were visualized using a GE chemiluminescence imaging system. The specific antibodies utilized in this study are detailed in Table S1.

### 2.7 Celigo cell count assay

Transfected RKO and HCT 116 cell lines in logarithmic growth phase were collected 48 hours post‐transfection, digested, resuspended in complete culture medium, and seeded into a 96‐well plate at a density of 2000 cells per well for further 24‐h incubation. The Celigo (Nexcelom) software was used to continuously scan the 96‐well plate for 5 consecutive days at the same time points. Finally, the cell proliferation curve was plotted based on the cell count values.

### 2.8 Colony formation assay

The RKO and HCT 116 cells, transduced with lentiviral vectors, were plated in a 6‐well plate with 2 mL of medium per well and cultured for 8 days, with the medium being refreshed every 3 days. Clones were observed and recorded using fluorescence microscopy. Following PBS washing, the cells were fixed with 4% paraformaldehyde and subjected to Giemsa staining (Dingguo, Shanghai, China). Images were taken, and the number of clones was calculated.

### 2.9 Cell Counting Kit‐8 (CCK‐8) assay

We harvested logarithmically growing RKO and HCT 116 cell lines, subjected them to trypsin digestion, and then resuspended them in complete culture medium. Afterward, the cells were seeded in a 96‐well plate at a density of 2000 cells per well. After a 24‐h incubation period, the cells were treated with 10 μL of CCK‐8 and incubated for an additional 4 h for 5 consecutive days. Subsequently, the optical density (OD) values at a wavelength of 450 nm were obtained using a microplate reader (Tecan Infinite) to create the cell proliferation curve over 5 days.

### 2.10 Flow cytometry

Apoptosis detection in RKO and HCT 116 cells was performed using flow cytometry after 5 days of transfection. In brief, the cells underwent digestion, followed by resuspension in complete medium and seeding into 6‐well plates for 24 h. After washing with precooled D‐Hanks at 4°C, the cells were resuspended in 1× binding buffer. Afterward, 10 μL of Annexin V‐APC (eBioscience) was added to the cell suspension and incubated for 10–15 min at room temperature in the absence of light. The assessment of cellular apoptosis was then carried out using a flow cytometer (Millipore).

### 2.11 Wound healing assay

Cells were resuspended in complete medium and then seeded at a rate of 50,000 cell per well into 96‐well plates until they reached a confluency of over 90%. Approximately 24 h later, the medium was changed to medium containing low serum, and a 96‐wounding replicator (VP scientific) was gently pushed upward towards the center of the bottom of the 96‐well plate to create a scratch. Subsequently, the plate was washed three times with serum‐free medium and then cultured in low serum medium. Cells in the same field were scanned and analyzed at 0 and 24 h with Cellomics (Thermo Fisher Scientific, Wilmington, USA). Images of the scratch were photographed under an inverted microscope (20×magnification; IX73; Olympus Corporation, Japan). Migration rate = (scratch area at 24 h − scratch area at 0 h)/ scratch area at 0 h.

### 2.12 Transwell assay

Briefly, the upper chamber of the Transwell (Corning) was initially filled with 100 μL serum‐free medium and allowed to incubate for 2 h. Subsequently, 100 μL of cell suspension containing 100,000 cells was carefully added to the upper chamber for cultivation, while the lower chamber received 600 μL of the same medium. Following overnight incubation, non‐migrating cells were gently eliminated using a cotton swab, while the migrating cells were immersed in 400 μL of staining solution for a duration of 5 min. The cells were washed multiple times with water, air‐dried, and finally subjected to microscopic imaging to determine the cell migration rate.

### 2.13 Xenograft tumor model

Six‐week‐old male BALB/c nude mice obtained from Jiangsu Jicui Yaokang Biological Technology Co., Ltd. were randomly assigned to the shCtrl group and the shSLC25A19 group, with five mice in each group. Briefly, RKO cells with or without SLC25A19 knockdown were subcutaneously injected into the nude mice at a concentration of 1 × 10^7^. Every 4 days starting from day 6, the tumor size and animal weight were analyzed. Tumor volume was calculated using the formula π/6  × length×2 tumor width. On day 22, the mice were sacrificed, and the solid tumors were photographed and weighed, and frozen in liquid nitrogen for Ki67 staining. All animal experiments were conducted following the approved protocols of the Institutional Animal Care and Use Committee of the School of Medicine of Shandong University (No. ECSBMSSDU2023‐2‐121).

### 2.14 Human phospho‐kinase array

Apoptosis‐related proteins was detected by human phospho‐kinase array kit (ARY003C, Bio‐Techne, China). Simply, RKO cells transfected with shSLC25A19 or shCtrl were lysed and the protein extract were collected. Subsequently, proteins were incubated with blocked antibody array membrane throughout the night at 4°C, followed by incubation with detection antibody cocktail for 2 h. After washing 1× Wash Buffer, membranes were incubated with array buffer containing diluted streptavidin‐ HRP for 30 min. The signals were detected by using enhanced chemiluminescence (ECL) (Amersham) and analyzed by Image J software.

### 2.15 Statistical analysis

Statistical analysis employed SPSS software, with GraphPad Prism utilized for generating statistical graphs. *T* tests were conducted for comparisons between two groups and comparison of multiple groups was performed one‐way ANOVA followed by post‐hoc test with Bonferroni correction. Mann–Whitney *U* test was used to assess the significance of SLC25A19 expression across pathological data. Spearman rank correlation analysis was performed to evaluate the positive or negative correlation between SLC25A19 expression and pathological parameters. All in vitro functional experiments were performed in biological triplicate. Data are shown as the mean ± standard deviation (SD), and statistical significance was determined with *p* < 0.05.

## 3 RESULTS

### 3.1 SLC25A19 is aberrantly expressed in CRC tissues and cells

**FIGURE 1:**
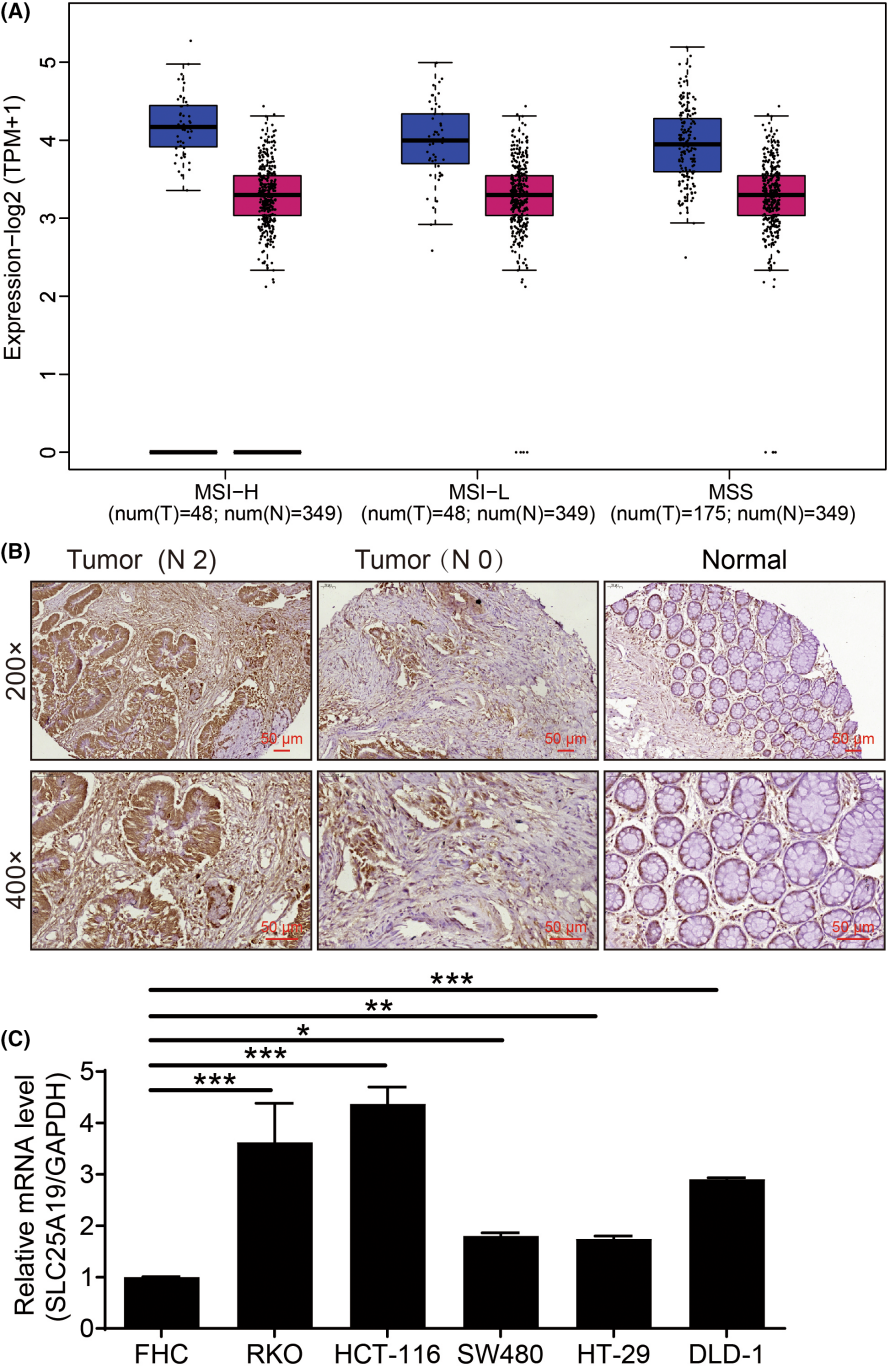
SLC25A19 was highly expressed in CRC tissues and cells. (A) The expression of SLC25A19 in CRC was determined by bioinformatics analysis using the TCGA database. (B) The expression of SLC25A19 was detected by IHC staining in 98 CRC tissues and 103 para‐carcinoma tissues. N: Lymphatic metastasis; N0: Without lymphatic metastasis. Scale bar: 50 μm. (C) The mRNA levels of SLC25A19 expression were measured by qRT‐PCR. *p < 0.05; **p < 0.01; ***p < 0.001.

To evaluate the roles of SLC25A19 in CRC, the expression patterns of SLC25A19 were initially determined in CRC tissues. Analysis of the TCGA database (http://gepia2.cancer‐pku.cn/#analysis) revealed significant upregulation of SLC25A19 expression in multiple tumor types, including colon adenocarcinoma (COAD), compared to normal tissues (Figure 1A and Figure S1). Additionally, the expression of SLC25A19 was validated in CRC tissues and para‐carcinoma tissues through IHC staining using a human tissue array. As demonstrated in Figure 1B, the expression of SLC25A19 was stronger in CRC tissues than in para‐carcinoma tissues. Importantly, high expression of SLC25A19 was observed in 47.2% of CRC tissues but only in 2.9% of para‐carcinoma tissues (Table 1) and was positively associated with age, gender, lymph node positivity, lymph node invasion, stage, and lymphatic metastasis (Tables 2 and 3), implying that the expression of SLC25A19 increased with the degree of malignancy in CRC patients. Furthermore, compared with FHC, SLC25A19 was highly expressed in the human CRC cell lines, notably in RKO and HCT 116 cells (Figure 1C). In short, SLC25A19 holds potential clinical value as a therapeutic target for the treatment of CRC.

**TABLE 1:** Expression patterns in colorectal cancer tissues and normal tissues revealed in immunohistochemistry analysis.

SLC25A19 expression	Tumor tissue		Normal tissue		*p* value
	Cases	Percentage	Cases	Percentage	
Low	52	53.1%	100	97.1%	<0.001
High	46	46.9%	3	2.9%	

**TABLE 2:** Relationship between SLC25A19 expression and tumor characteristics in patients with colorectal cancer.

Features	No. of patients	SLC25A19 expression		*p* value
		Low	High	
All patients	98	52	46	
Age (years)				0.072
≤61	50	31	19	
>61	48	21	27	
Gender				0.019
Male	57	36	21	
Female	41	16	25	
Number of lymph nodes (*N*)				0.308
≤16	50	24	26	
>16	48	28	20	
Lymph node positive (*N*)				0.001
=0	50	35	15	
>0	48	17	31	
Lymph node invasion				0.001
No	50	35	15	
Yes	48	17	31	
Differentiation				0.856
Low	10	6	4	
Medium	85	44	41	
High	3	2	1	
Stage				0.023
I	11	8	3	
II	36	23	13	
III	43	17	26	
IV	8	4	4	
T Infiltrate				0.189
T1	2	2	0	
T2	14	9	5	
T3	48	25	23	
T4	34	16	18	
Lymphatic metastasis (*N*)				0.009
N0	50	34	16	
N1	33	11	22	
N2	15	7	8	
Vascular invasion				0.901
0	94	50	44	
1	4	2	2	
Metastasis				0.857
M0	90	48	42	
M1	8	4	4	

**TABLE 3:** Relationship between SLC25A19 expression and tumor characteristics in patients with colorectal cancer.

		SLC25A19
Gender	Spearman correlation	0.239
	Significance (two‐tailed)	0.018
	*N*	98
Lymph node positive	Spearman correlation	0.346
	Significance (two‐tailed)	*p* < 0.001
	*N*	98
Lymph node invasion	Spearman correlation	0.346
	Significance (two‐tailed)	*p* < 0.001
	*N*	98
Lymphatic metastasis (N)	Spearman correlation	0.265
	Significance (two‐tailed)	0.008
	*N*	98
Stage	Spearman correlation	0.231
	Significance (two‐tailed)	0.022
	*N*	98

### 3.2 Construction of SLC25A19 knockdown cell models in CRC cells

**FIGURE 2:**
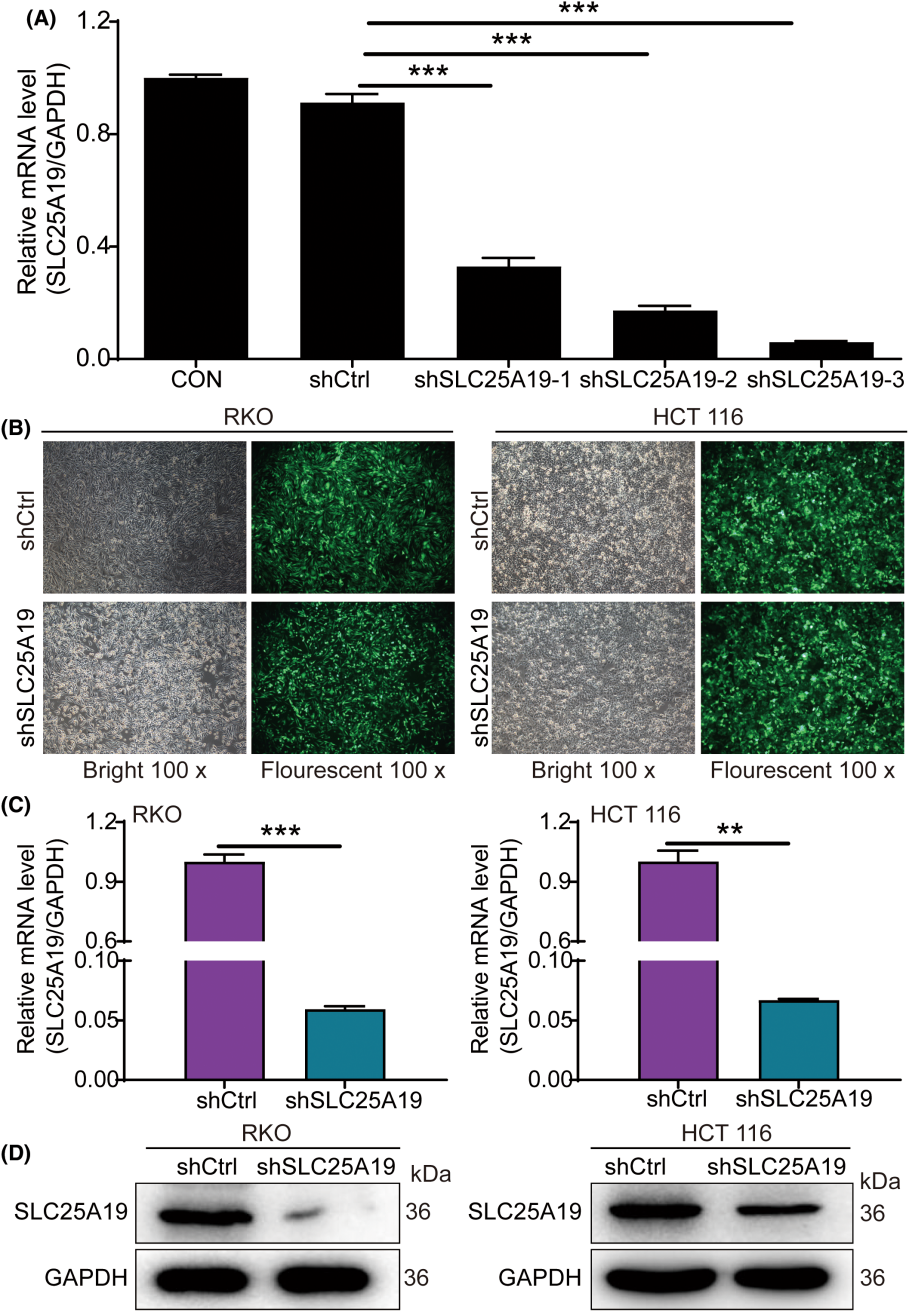
Construction of stable SLC25A19 knockdown CRC cell lines. (A) The interference efficiencies of three SLC25A19 shRNA sequences. (B) Fluorescence imaging of transfected RKO and HCT 116 cells. (C, D) SLC25A19 knockdown was validated in RKO and HCT 116 cells by qPCR analysis (C) and western blotting (D). Data were shown as mean ± standard deviation (SD). **p < 0.01; ***p < 0.001.

To determine the impact of SLC25A19 in CRC cells, SLC25A19‐depleted RKO and HCT 116 cell lines were established through lentivirus infection. The qPCR results revealed that shSLC25A19‐3 exhibited a greater knockdown efficiency in HCT 116 cells than shSLC25A19‐1 and shSLC25A19‐2 (Figure 2A) and was selected for use in subsequent experiments. After infection with shCtrl or shSLC25A19 for 72, fluorescence of RKO and HCT 116 cells was visualized under a microscope, revealing >80% transfection efficiency for both cell lines (Figure 2B). Furthermore, the expression of SLC25A19 in the two cell lines transfected with shSLC25A19 was significantly suppressed at both mRNA and protein levels (Figure 2C,D), suggesting successful knockdown of SLC25A19.

### 3.3 SLC25A19 knockdown suppressed the malignant phenotype of CRC cell lines

**FIGURE 3:**
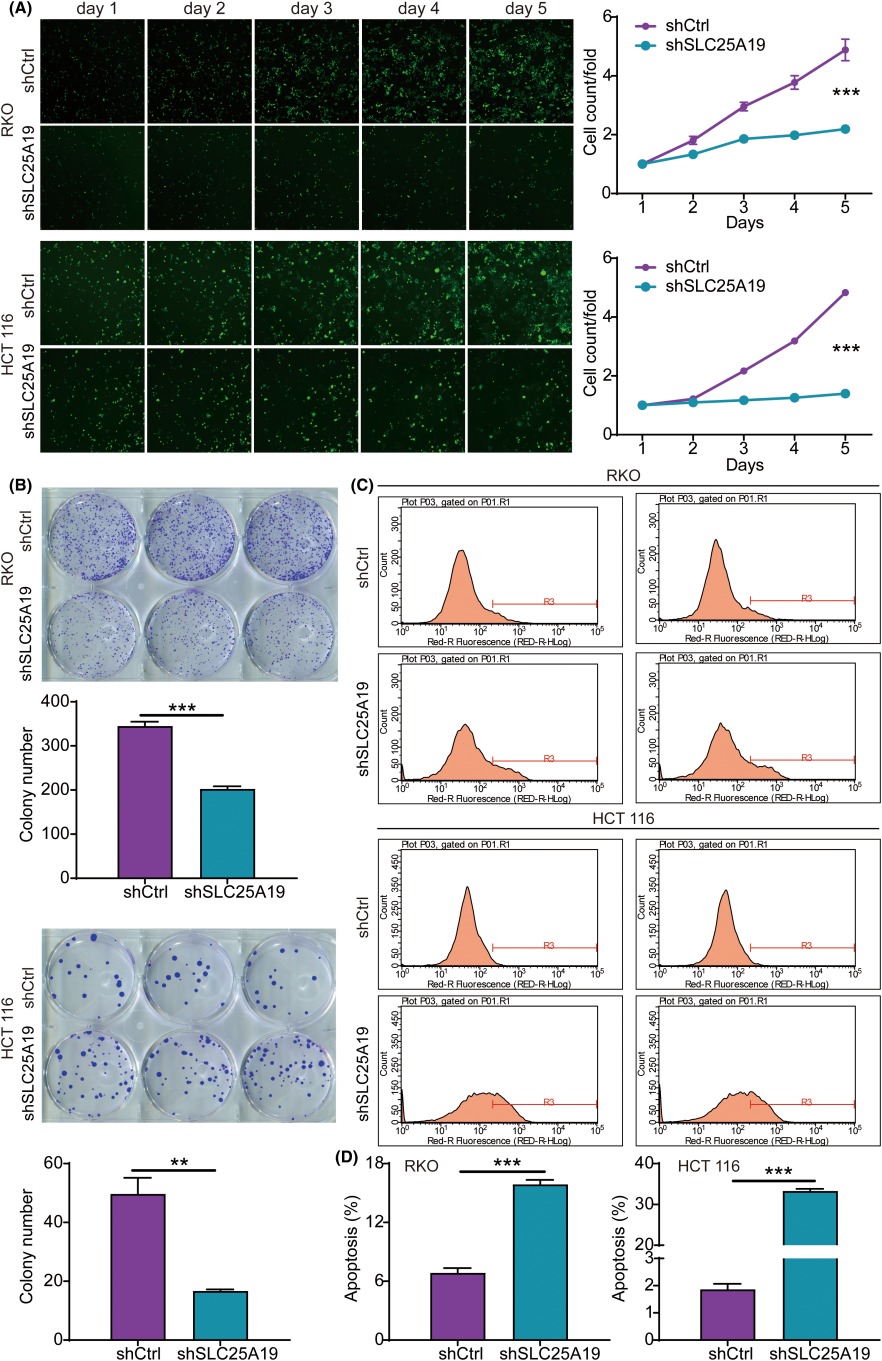
SLC25A19 knockdown inhibited the proliferation of CRC cells and induced apoptosis. (A) A Celigo cell counting assay was used to evaluate the proliferation of RKO and HCT 116 cells. (B) Clone formation assays were performed to assess the cloning ability of RKO and HCT 116 cell lines following SLC25A19 knockdown. (C, D) The apoptosis of RKO and HCT 116 cells was detected by flow cytometry. Experiments were performed in triplicate. Data were shown as mean ± standard deviation (SD). **p < 0.01, ***p < 0.001.

Next, the functional impact of SLC25A19 on the proliferation, apoptosis, and migration of CRC cells were investigated using stable SLC25A19 knockdown cell lines. The Celigo cell counting assay results showed a significant weakening of the proliferative ability of RKO and HCT 116 cells upon SLC25A19 downregulation, in comparison to the shCtrl group (Figure 3A). Additionally, colony formation was also diminished in both cell lines after SLC25A19 silencing (Figure 3B). Furthermore, flow cytometry analysis revealed a substantial increase in the apoptotic proportion of RKO and HCT 116 cells in the shSLC25A19 group in comparison to the shCtrl group (Figure 3C,D). Therefore, these results provided evidence that SLC25A19 knockdown may suppress proliferation and induce apoptosis in CRC cell lines.

**FIGURE 4:**
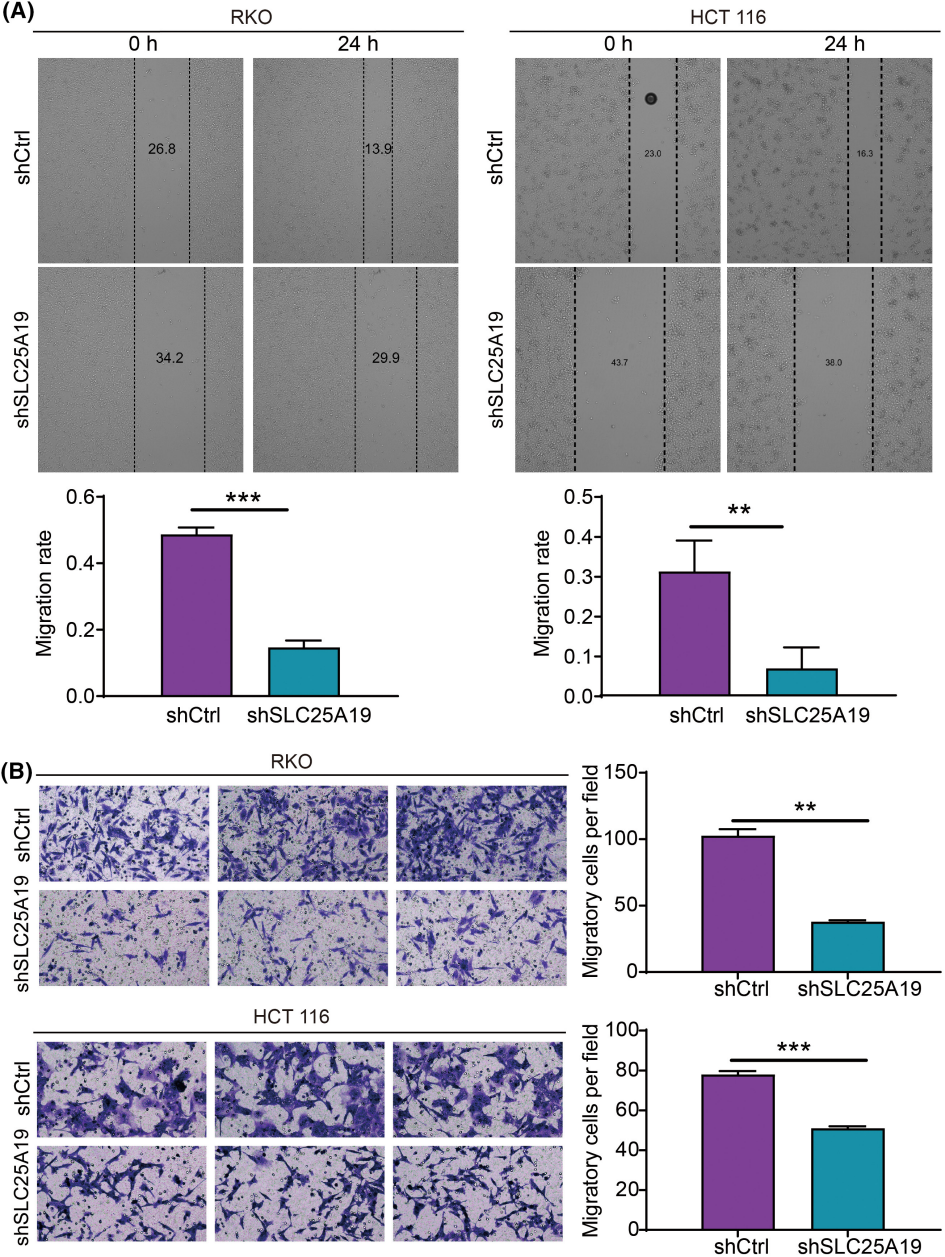
SLC25A19 knockdown inhibited the migration of CRC cells. (A) The migration of RKO and HCT 116 cells with or without SLC25A19 knockdown was assessed by wound healing assay. (B) Transwell assays were performed to validate the effect of SLC25A19 knockdown on the migration of RKO and HCT 116 cells. All experiments were performed in triplicate. Data were shown as mean ± standard deviation (SD). **p < 0.01; ***p < 0.001.

Given the association of SLC25A19 with metastasis and the high susceptibility of CRC to metastasis, we examined the impact of SLC25A19 on the migratory ability of RKO and HCT 116 cells using wound healing and transwell assays. Following the knockdown of SLC25A19, the migration rates of RKO cells (24 h) showed a decrease of 18%, while HCT 116 cells (24 h) exhibited a decrease of 55% compared to the shCtrl group (Figure 4A). Additionally, the transwell assay results further demonstrated a noticeable attenuation in the migratory capacity of both cell lines upon SLC25A19 knockdown (Figure 4B). The collective findings suggest that the knockdown of SLC25A19 exerts an inhibitory effect on the malignant phenotype of CRC cells.

### 3.4 SLC25A19 was crucial for tumor growth in CRC mice in vivo

**FIGURE 5:**
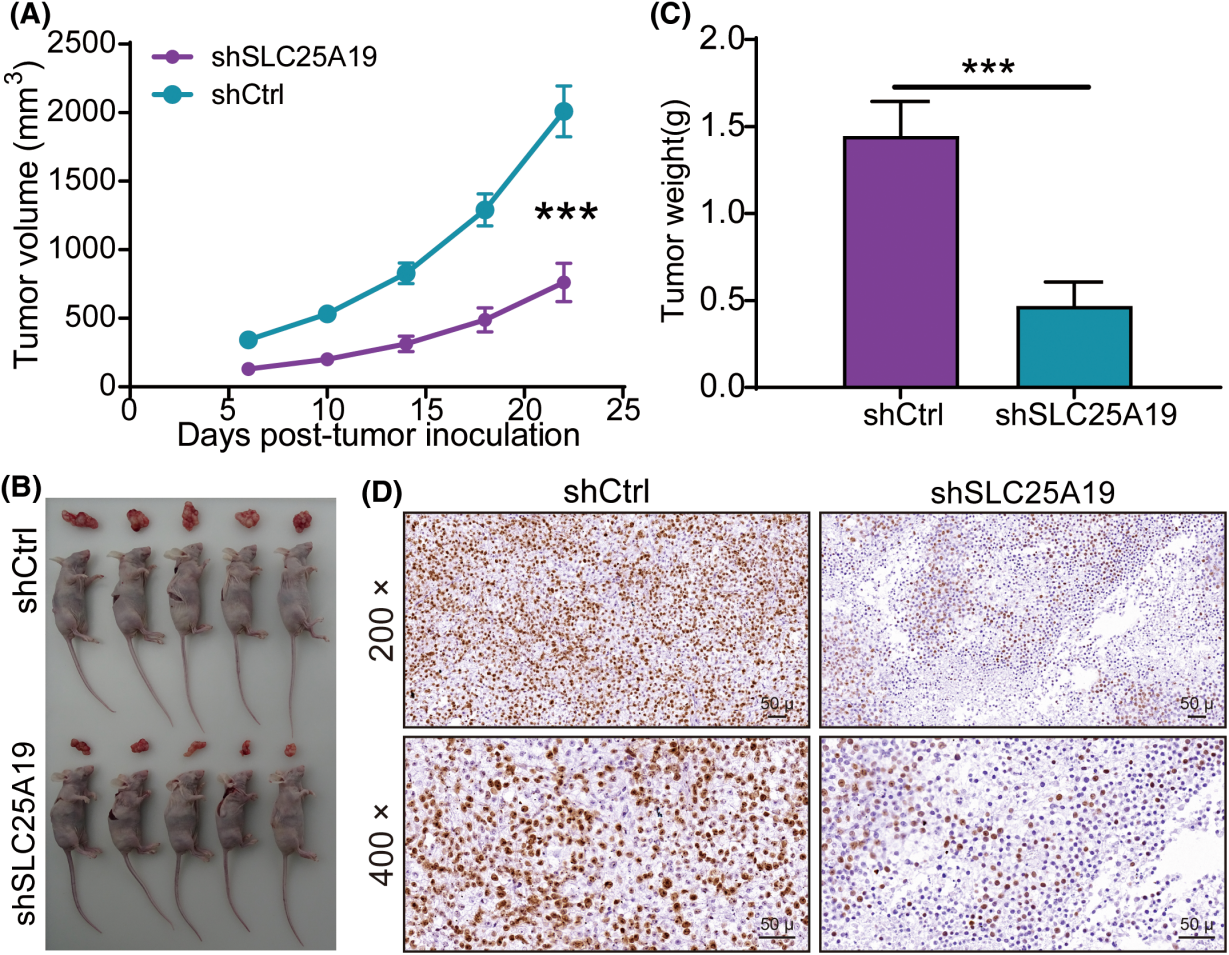
SLC25A19 knockdown significantly inhibited tumor growth in vivo. (A) The tumor growth curves of in vivo tumor volumes. (B) Pictures of mice and tumors in both groups. (C) Weight of tumors in nude mice. (D) Representative IHC staining images for Ki67 were taken at 200× and 400× magnification. Scale bars: 50 μm. Data were shown as mean ± standard deviation (SD). ***p < 0.001.

Next, we established mouse xenograft models to assess the impact of SLC25A19 on CRC tumor growth in vivo by subcutaneously injecting RKO cells transfected with shCtrl or shSLC25A19 into nude mice. The tumor growth curves demonstrated a reduced rate of growth (Figure 5A), as evidenced by smaller tumor sizes (Figure 5B) and a significantly lighter mean tumor weight in the shSLC25A19 group compared to the shCtrl group (Figure 5C). Upon sacrificing the mice, the isolated tumor tissues were subjected to IHC staining to identify Ki‐67 expression, an indicator of proliferation. Consistent with the in vitro proliferation assay, the tumors from the shSLC25A19 group contained fewer Ki‐67‐positive cells than those from the shCtrl group (Figure 5D), confirming that SLC25A19 may contribute to tumor proliferation in vivo.

### 3.5 SLC25A19 contributes to CRC cell growth and apoptosis through p53

**FIGURE 6:**
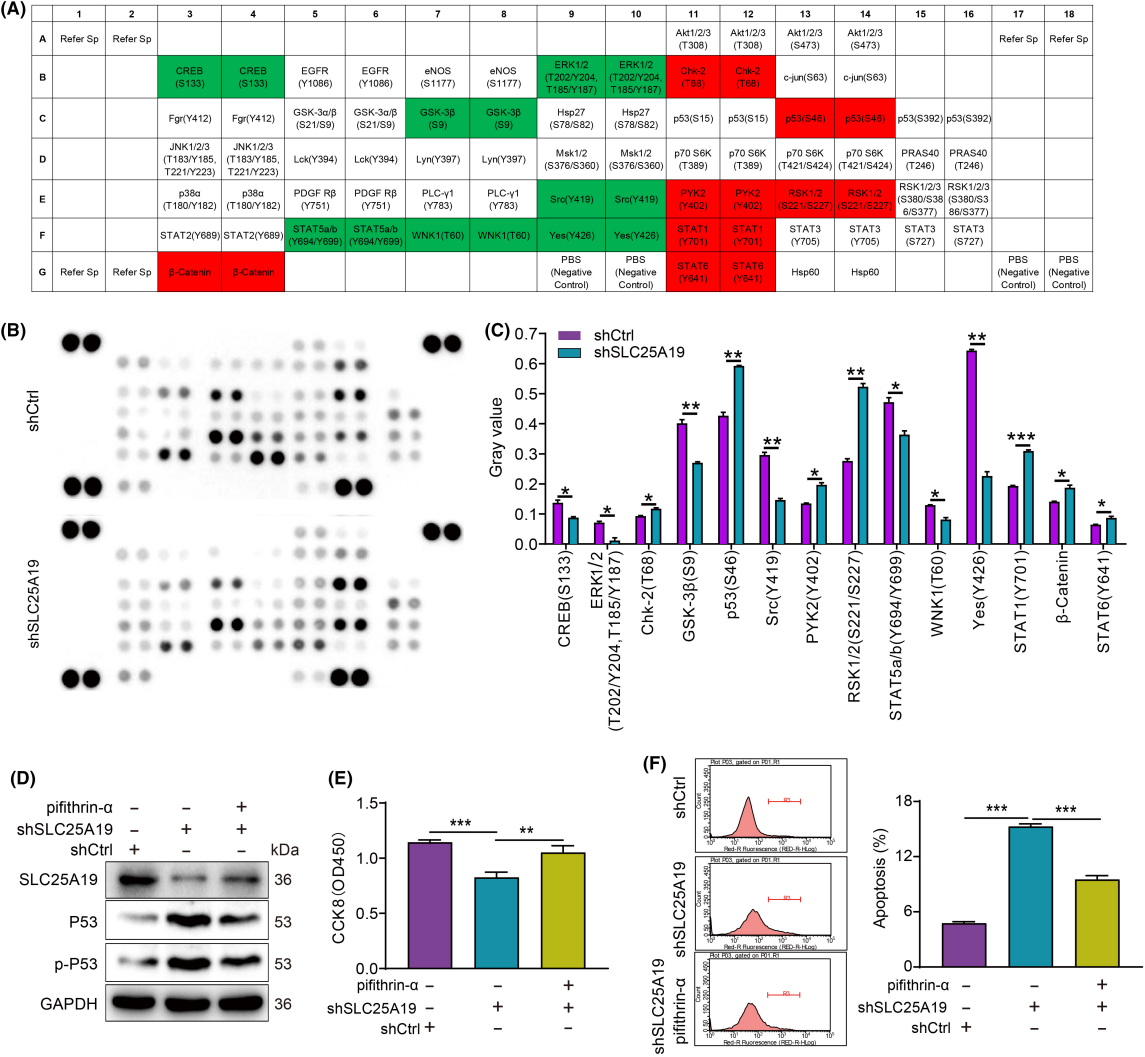
SLC25A19 regulated cell proliferation and apoptosis through p53. (A–C) A human phosphokinase array assay was used to examine the phosphorylation levels of cancer‐associated proteins following SLC25A19 knockdown. Representative images of the phosphokinase array membranes for shCtrl and shSLC25A19 groups. Each pair of dots represents a specific phosphorylated kinase, with signal intensity corresponding to the level of phosphorylation. Red bars indicate upregulation, while green bars indicate downregulation (A, B). Quantification of relative phosphorylation levels (C). (D) Western blotting was used to detect the expression of p53 and phosphorylated p53 in RKO cells in the absence or presence of the p53 inhibitor pifithrin‐α. (E) The proliferation of SLC25A19 knockdown RKO cells with or without pifithrin‐α treatment was detected by CCK8 assay. (F) The apoptosis of RKO cells was determined by flow cytometry. All in vitro functional experiments were performed in triplicate. Data were shown as mean ± standard deviation (SD). *p<0.05; **p < 0.01; ***p < 0.001.

To clarify the molecular mechanisms by which SLC25A19 regulates CRC progression, we examined the phosphorylation level of key proteins in cancer‐related pathways by using the human phospho‐kinase array. As depicted in Figure 6A, SLC25A19 knockdown led to a notable increase in the phosphorylation levels of p53, RSK1/2, and STAT1, while also causing marked reductions in GSK‐3β, Src, and Yes compared to the shCtrl group (Figure 6A–C). Furthermore, Western blot results indicated an upregulation of p53 and phosphorylated p53 in SLC25A19 knockdown RKO cells, which was partially reversed by the p53 inhibitor pifithrin‐α (Figure 6D). Notably, inhibiting p53 reversed the suppressive effect of SLC25A19 knockdown on RKO cell proliferation (Figure 6E and Figure S1). Furthermore, treatment with pifithrin‐α attenuated the proapoptotic effect of SLC25A19 knockdown (Figure 6F). Collectively, these results indicated that SLC25A19 regulated cell growth and apoptosis in CRC through the p53 pathway.

## 4 DISCUSSION

The SLC superfamily is one of the largest groups of membrane transport proteins in humans, consisting of 65 families and over 400 SLC transporters.^16^, ^17^, ^18^ Tumorigenesis is a complex process that involves multiple steps leading to the malignant transformation of normal cells.^19^, ^20^ The SLC superfamily has been increasingly associated with crucial roles in tumorigenesis, encompassing a wide range of cancer‐related processes such as proliferation, apoptosis, invasion, and metastasis, as well as resistance to chemotherapy.^21^ What's more, certain SLC transporters have shown promise as novel targets for enhancing chemotherapy sensitivity and surmounting drug resistance.^22^ Therefore, manipulating the expression of SLCs offers promising avenues for diagnosing, treating, or predicting the prognosis of cancer.

SLC25A19 is one of the SLC superfamily members of interest recently, and its functional studies are still in their infancy. Research has implicated SLC25A19 in the progression of various diseases, including mitochondrial diseases,^23^ neurological disorders,^24^ and cancer.^14^, ^25^, ^26^ For instance, SLC25A19 deficiency might result in bilateral striatal degeneration and progressive polyneuropathy.^27^ In prostate cancer, the decreased expression of SLC25A19 plays a role in regulating the tricarboxylic acid cycle metabolism by restricting the availability of the cofactor thiamine pyrophosphate.^25^ Previous studies also showed that upregulation of UCHL5 might drive bladder cancer carcinogenesis by triggering the expression of SLC25A19.^26^ Nevertheless, the biological roles of SLC25A19 in cancer cellular properties and tumor growth have not been reported thus far.

In our study, we delved into the investigation of SLC25A19 expression in CRC and its potential impact on tumor progression. Upon analyzing the protein expression of SLC25A19 in CRC tumor tissues and adjacent noncancerous tissues, we observed a significant upregulation of SLC25A19 in CRC. Additionally, we noted a positive correlation between the upregulation of SLC25A19 and age, sex, lymph node positivity, lymph node invasion, stage, and lymphatic metastasis, indicating that SLC25A19 could potentially serve as a novel therapeutic target for CRC treatment.

Dysregulation of proliferation, apoptosis and metastasis are critical malignancy hallmarks and major contributors to cancer‐related mortality.^28^, ^29^, ^30^ Normally, suppressing cell proliferation and migration, along with inducing cell apoptosis are thought to be an effective approach for arresting cancer development.^31^ Then, in this study, a series of loss‐of‐function experiments were conducted to explore the consequence of SLC25A19 knockdown on the malignant phenotype of CRC cells, including proliferation, apoptosis, and migration. We found that SLC25A19 deletion significantly reduced proliferation and colony formation, accelerated apoptosis, and severely impaired cell migration in vitro. Subsequently, a xenograft tumor model was constructed to validate the inhibitory effects of SLC25A19 knockdown. It showed that downregulated SLC25A19 could delay tumor formation and retard tumor growth. Ki67, a biomarker of in vivo proliferation,^32^ is associated with prognosis, progression, and metastatic risk in cancers.^33^ The expression of Ki67 in tumor sections of the shSLC25A19 group was weaker compared to the shCtrl group in mice, which aligns well with the in vitro results. Collectively, these results suggested that downregulation of SLC25A19 exerted an inhibitory effect on the aggressive behaviors of CRC cells.

Notably, p53 is a proapoptotic protein that is implicated in cell growth, apoptosis, and DNA repair.^34^ Strong evidence has accumulated that p53 plays a critical inhibitory role in cancer progression.^35^, ^36^ Our further mechanistic exploration of the study revealed that SLC25A19 knockdown promoted the phosphorylation and expression of p53. Interestingly, pifithrin‐α, a p53 inhibitor, was able to attenuate these influences of SLC25A19 deficiency. In line with this, functional experiments demonstrated that the SLC25A19 deletion‐mediated pro‐proliferative and anti‐apoptotic effects were partially reversed in the presence of pifithrin‐α. These findings indicated that SLC25A19 influences cell proliferation and apoptosis through the p53 pathway.

In this study, SLC25A19 knockdown produced strong inhibitory effects on cancer‐related phenotypes and tumor growth, highlighting SLC25A19's importance in CRC progression. However, there are also some limitations of the study. First, the experimental tissue sample size is limited and needs to be expanded in future research. Additionally, the current research only provides a preliminary understanding of SLC25A19's expression and function in CRC. The exact mechanisms, particularly the potential regulatory relationship and mechanism between SLC25A19 and p53, remain unclear. Further studies using mutant or knockout p53 cell lines are essential to determine whether p53 is integral to SLC25A19's regulatory role in CRC progression.

In conclusion, a novel CRC promotor, SLC25A19, was identified and functionally studied. Functional analysis revealed that knockdown of SLC25A19 effectively suppressed CRC cell proliferation and migration, induced apoptosis in vitro, and attenuated tumor growth in vivo. Furthermore, SLC25A19 deficiency inhibited the malignant behaviors of CRC cells through the p53 pathway. These results strongly suggested that SLC25A19 has the potential to serve as both a biomarker and a therapeutic target for CRC.

## AUTHOR CONTRIBUTIONS

**Jinbo Jiang:** Data curation (equal); formal analysis (equal); writing – original draft (equal). **Xuemei Li:** Data curation (equal); formal analysis (equal). **Jiayong Wang:** Data curation (equal); formal analysis (equal). **Shaofei Chen:** Formal analysis (equal); methodology (equal); software (equal). **Lingjuan Chen:** Conceptualization (equal); supervision (equal); writing – review and editing (equal).

## FUNDING INFORMATION

This work was supported by Shandong Provincial Natural Science Foundation, China (No. ZR2019MH071), Bethune‐Cancer Radiotherapy Translational Medicine Research Fund of China (Grant No. flzh202117) and the Beijing Kechuang Medical Development Foundation Fund of China (Grant No. KC2021‐JX‐0186‐31).

## CONFLICT OF INTEREST STATEMENT

The authors have no conflicts of interest to declare.

## ETHICS STATEMENT

The study was approved by Institutional Animal Care and Use Committee of the School of Medicine of Shandong University (No. ECSBMSSDU2023‐2‐121). All experimental procedures in this study were conducted in strict adherence to the applicable guidelines and regulations. The reporting of this study is in accordance with the ARRIVE guidelines (https://arriveguidelines.org), which provide recommendations for the transparent and comprehensive reporting of animal research.

## Supporting information


Data S1:

## Data Availability

The data obtained from the current study have been incorporated into the figures and/or tables presented in this article.
