# Supplementary material for: SLC25A19 drives colorectal cancer progression by regulating p53
Source: Cancer Med. 2024 Sep 30;13(18):e70253. doi: 10.1002/cam4.70253 (PMC11440145; doi:10.1002/cam4.70253)
Supplement: Supplementary file 1 — Data S1: [file CAM4-13-e70253-s001.docx]

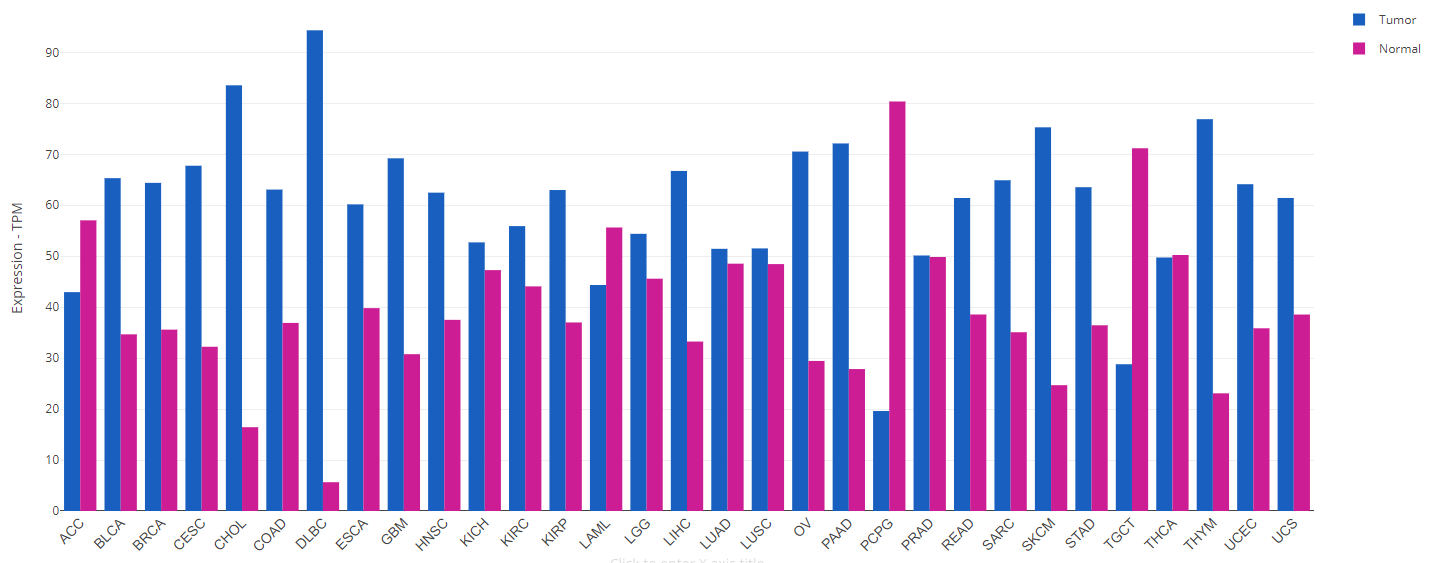


Figure S1. Expression profile of SLC25A19 in multiple cancer types.


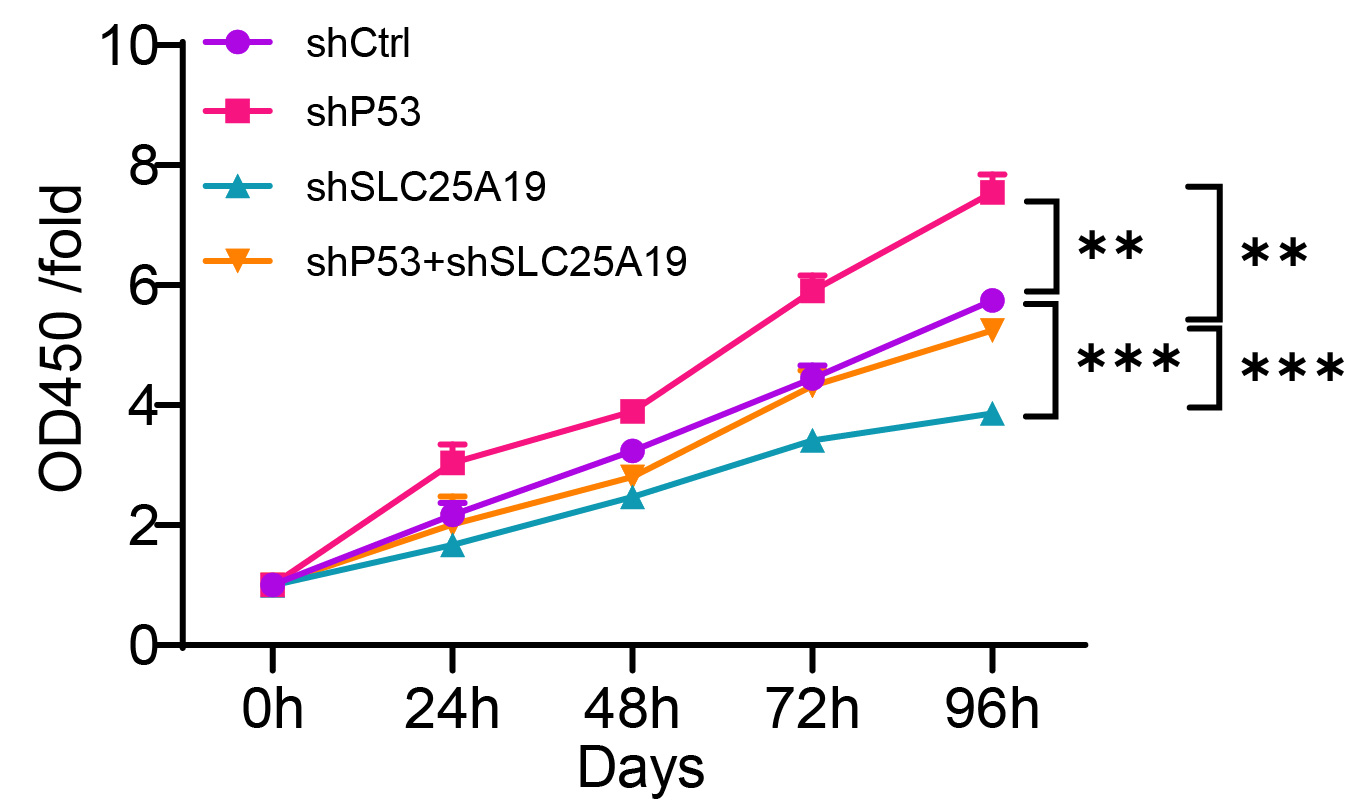


Figure S2. The CCK-8 assay was used to assess the proliferation of SLC25A19 knockdown RKO cells, with or without p53 knockdown.

**Table S1** Antibodies applied in Western blot of this study.

| Primary antibody | Size/kDa | Diluted multiples | Source | Company | Catalog No. |
| --- | --- | --- | --- | --- | --- |
| SLC25A19 | 36 | 1:500 | Rabbit | Abcam | ab190228 |
| P53 | 53 | 1:2000 | Rabbit | Proteintech | 10442-1-AP |
| p-P53 | 53 | 1:1000 | Rabbit | Abcam | ab76242 |
| GAPDH | 36 | 1:30000 | Mouse | Proteintech | 60004-1-lg |
| Secondary antibody |  | Diluted multiples |  | Company | Catalog No. |
| Goat Anti-Rabbit |  | 1:3000 |  | Beyotime | A0208 |
| Goat Anti-Mouse |  | 1:3000 |  | Beyotime | A0216 |
